# Evidence of racial differences in peripheral blood pressure, central haemodynamics and arterial stiffness between young Black and White women

**DOI:** 10.1113/EP092929

**Published:** 2025-07-26

**Authors:** Michele N. D'Agata, Elissa K. Hoopes, Alexs A. Matias, Mackenzie L. Rattigan, Freda Patterson, Melissa A. Witman

**Affiliations:** ^1^ Department of Kinesiology and Applied Physiology University of Delaware Newark Delaware USA; ^2^ Department of Health Behavior and Nutrition Sciences University of Delaware Newark Delaware USA

**Keywords:** blood pressure, pulse wave analysis, pulse wave velocity, racial disparity

## Abstract

Hypertension diagnosed via peripheral (brachial) blood pressure (pBP) is a strong independent predictor of overt cardiovascular disease (CVD). However, central (aortic) blood pressure (cBP), which is influenced by arterial stiffness, may be more strongly associated with CVD risk. Young Black women (BLW) demonstrate higher pBP than White women (WHW), but investigations of racial differences in central haemodynamics and arterial stiffness in young women are lacking. We assessed pBP, central haemodynamics and arterial stiffness in young, non‐hypertensive BLW and WHW. We hypothesized that pBP, central haemodynamics (cBP, augmentation pressure (AP), augmentation index normalized to a heart rate of 75 beats per minute (AIx75), arterial wave reflections), and arterial stiffness (carotid–femoral pulse wave velocity (cf‐PWV)) would be higher in BLW. Under standardized resting conditions, supine brachial pBP was measured, and central haemodynamics were estimated via pulse wave analysis using partial cuff inflation. cf‐PWV was assessed via simultaneous carotid artery applanation tonometry and partial cuff inflation over the femoral artery. Participants were young, apparently healthy women who self‐identified their race as Black (BLW: *n* = 44) or White (WHW: *n* = 40). Systolic pBP (*P* = 0.04) and diastolic pBP (*P *< 0.01) were higher among BLW. Systolic cBP (*P *< 0.01), diastolic cBP (*P *< 0.01), heart rate (*P *< 0.001), AP (*P* = 0.02), AIx75 (*P *< 0.001), arterial wave reflection magnitude (*P* = 0.40) and cf‐PWV (*P* = 0.04) were all higher among BLW. Findings demonstrate elevations in pBP, central haemodynamics and arterial stiffness in young BLW versus WHW. Central haemodynamics and arterial stiffness may be promising targets in the early assessment of CVD risk in young BLW.

## Abstract (highlights)

- **What is the central question of this study?**
  Are there racial differences in peripheral blood pressure, central haemodynamics and arterial stiffness between young, generally healthy, non‐hypertensive Black and White women?
- **What is the main finding and its importance?**
  Findings demonstrate increased peripheral blood pressure (BP), central BP, augmentation index, and arterial stiffness in young Black women compared with White women.  It is well‐documented that Black women have the highest prevalence of hypertension, a major but modifiable risk factor for cardiovascular disease (CVD). Increased central BP and arterial stiffness are strong independent risk factors for CVD and findings indicate that these may also be important intervention targets for the prevention of CVD in Black women.

## 1 INTRODUCTION

Arterial hypertension (HTN) is a strong independent predictor of cardiovascular disease (CVD) and affects around 15% of young women of reproductive age in the USA (Weng et al., 2023). However, marked racial differences in HTN prevalence are observed early in life, demonstrating disproportionately higher rates of HTN in young non‐Hispanic Black women (BLW) compared with non‐Hispanic White women (WHW) (22.3% vs. 14.4%, respectively) (Weng et al., 2023). By mid‐life, the cumulative incidence of HTN in BLW is nearly twice that of WHW (Thomas et al., 2018) and the average age at onset occurs between 5.4 and 7.7 years earlier among BLW compared with WHW (Reeves et al., 2022).

Although measurement of peripheral (brachial) blood pressure (pBP) is clinically relevant and important for the diagnosis of arterial HTN (Flack & Adekola, 2020), central (aortic) blood pressure (cBP) may be a better prognostic indicator of future CVD (McEniery et al., 2014). Elevations in cBP have been shown to relate to target organ damage, independent of pBP (Wang et al., 2009). Moreover, other aspects of central haemodynamics including arterial wave reflections and augmentation index (AIx) have been found to independently predict future cardiovascular events (Vlachopoulos et al., 2010). Notably, these cardiovascular parameters are closely interrelated; for example, arterial stiffness is bidirectionally associated with arterial pressure, and both can contribute to the timing of arterial wave reflections to influence AIx and cBP (Kim, 2023). Large population‐based studies demonstrate a higher AIx among Black compared with White adults independent of age and several other demographic variables; however cBP values were not reported (Chirinos et al., 2011; Morris et al., 2013). One study conducted in young men reported higher systolic cBP and higher AIx in Black men compared with White men, despite similar pBP, suggesting that elevations in cBP may precede elevations in pBP (Heffernan et al., 2008). Still, studies evaluating central haemodynamics in young BLW are lacking, despite the disproportionately high burden of HTN and CVD in this population (Martin et al., 2024).

Arterial stiffness is an important predictor of CVD outcomes (Kim & Kim, 2019) and can contribute to elevations in both pBP and cBP (Najjar et al., 2008; Nichols et al., 2008). For example, in addition to elevated cBP, arterial stiffness assessed as carotid–femoral pulse wave velocity (cf‐PWV) was higher in young Black men compared with White men (Heffernan et al., 2008). A separate study reported no effect of race or sex on cBP among young Black and White men and women, although pBP and cf‐PWV were lower in women compared with men, independent of race (Yan et al., 2014). In contrast, a meta‐analysis reported increased arterial stiffness assessed by cf‐PWV in generally healthy Black compared with White adults, although differences were larger among males (Buie et al., 2019). Studies evaluating the effect of race on central haemodynamics and arterial stiffness exclusively in young healthy women are lacking, as most previous studies have been conducted in men or have not stratified findings by sex.

The purpose of this study was to test for racial differences in pBP, central haemodynamics and arterial stiffness in young, otherwise healthy, non‐hypertensive BLW and WHW. We hypothesized that pBP, central haemodynamics including cBP, arterial wave reflections, and AIx, and arterial stiffness would be increased in BLW compared with WHW.

## 2 METHODS

### 2.1 Ethical approval

All procedures performed in this study were approved by the University of Delaware Institutional Review Board (IRB study No. 1957713, 1704969 and 1288814) and were conducted in accordance with the *Declaration of Helsinki*, except for registration in a database. Written informed consent was obtained from all participants prior to participation.

### 2.2 Participants and experimental procedures

Participants were recruited from the University of Delaware and the surrounding Newark, DE region and included apparently healthy cisgender women between the ages of 18 and 29 years who self‐identified their race as Black or White. As these data were collected as part of three larger studies focused on cardiometabolic health outcomes in young adults, individuals were excluded for the following: a history of any major chronic diseases or conditions, including cardiovascular, renal, metabolic, autoimmune or cancerous conditions; a recent history of infection with (<60 days) or vaccination from (<14 days) coronavirus disease 2019; seated resting BP >140 or >90 mmHg; use of any medication known to affect BP or significantly alter cardiovascular physiology; body mass index (BMI) >35 kg/m^2^; current tobacco use (≥1 cigarette in the last month); diagnosis of a sleep disorder (e.g. insomnia, restless leg syndrome, sleep apnoea); diagnosis of depression; use of any supplements or medications for sleep; current night‐shift work; women who were currently pregnant or breastfeeding; women who reported being perimenopausal or post‐menopausal. Menstrual cycle was not controlled for during testing.

Screening procedures required participants to complete a review of personal and parental medical history and they were asked to report on current medication use, gynaecological and menstrual history, hormonal contraceptive use and educational attainment. Seated resting BP was assessed in triplicate for screening purposes only (Omron 5 Series, BP7200, Omron Healthcare, Hoffman Estates, IL USA), height and weight were measured for the calculation of BMI, and body fat percentage was determined via bioelectrical impedance analysis (Tanita TBF‐300A, Arlington Heights, IL, USA). Eligible participants also underwent fasted intravenous blood sampling for the clinical assessment of blood lipids and blood glucose (Study No. 1704969: Quest Diagnostics, Inc., Philadelphia, PA, USA; Study No. 1957713: LabCorp Testing Services, Burlington, NC USA).

### 2.3 Pulse wave analysis

Participants returned to the laboratory on a separate day during the morning hours (between 07.00 and 11.00 h) following an overnight fast. Participants were asked to refrain from caffeine for ≥12 h, from alcohol, exercise and vitamins/supplements for ≥24 h, and from over‐the‐counter medications or anti‐inflammatory drugs for ≥72 h prior to the visit. Participants reported if they were currently menstruating or at the placebo phase of their oral contraceptives on the morning of testing. After 15 min of quiet rest in a dim‐lit room, supine measurements were performed in triplicate and averaged using the SphygmoCor Xcel System (AtCor Medical, Sydney, NSW, Australia). Using a standard BP cuff, systolic and diastolic pBP were measured in the left brachial artery, automatically followed by partial cuff inflation to estimate central haemodynamics from brachial artery pressure waveforms using pulse‐wave analysis (PWA) (Hwang et al., 2014). Brachial waveforms were used to generate central aortic pressure waveforms by applying proprietary digital signal processing and a generalized transfer function (Hwang et al., 2014). PWA measurements of systolic and diastolic cBP, central pulse pressure (PP), heart rate (HR), augmentation pressure (AP), AIx, AIx normalized to a HR of 75 beats/min (AIx75), forward pulse height, reflected pulse height, and reflection magnitude (RM; the ratio of the reflected pulse height and forward pulse height) were recorded. Pulse pressure amplification was derived from peripheral and central pulse pressures by dividing brachial pulse pressure by aortic pulse pressure (Papaioannou et al., 2010).

### 2.4 Carotid–femoral pulse wave velocity

Immediately following PWA, supine cf‐PWV was assessed using the SphygmoCor Xcel. On the left side of the body carotid pulse waves were recorded via applanation tonometry, and femoral pulse waves were simultaneously recorded via partial cuff inflation over the femoral artery. cf‐PWV was calculated as the ratio of the corrected distance between the carotid and femoral pulse measuring sites to the time delay between the carotid and femoral pulse waves (Hwang et al., 2014). Distance was measured with a non‐stretchable measuring tape from the (1) suprasternal notch to the carotid site, (2) femoral artery at the inguinal ligament to the proximal edge of the thigh cuff, and (3) suprasternal notch to the proximal edge of the thigh cuff (Hwang et al., 2014). Distances (1) and (2) were subtracted from distance (3) and used in the calculation of cf‐PWV (m/s). The average of three high‐quality cf‐PWV recordings was used (Hwang et al., 2014). A high‐quality recording was determined by the SphygmoCor Xcel system based on Quality Control (QC) indices and defined as an overall quality value ≥75%, according to manufacturer's guidelines.

### 2.5 Statistical analyses

Normality testing was performed using D'Agostino–Pearson tests to determine parametric or non‐parametric statistical testing. An independent samples Student's *t*‐test (parametric) or a Mann–Whitney *U*‐test (non‐parametric) was used to assess group differences in continuous variables. Fisher's exact test was used to assess group differences in categorical variables. Effect size was assessed using Cohen's *d*, with *d* ≥ 0.80 indicating a large effect. Statistical significance was set a priori at *P* < 0.05 (GraphPad Prism, Version 10.2, GraphPad Software, Boston, MA, USA). Data are presented as means ± standard deviation for continuous variables or *n* (%) for categorical variables.

## 3 RESULTS

Eighty‐four women (44 BLW, 40 WHW) completed study assessments. Participant characteristics are in Table 1. No differences were observed for age, BMI, body fat percentage, parent history of HTN and most clinical blood values. Blood triglycerides were significantly lower in BLW (*P *< 0.001), although values for both groups were within normal clinical ranges. All but two participants (*n* = 1 BLW and 1 WHW) were nulliparous. Forty‐four women were naturally cycling (29 BLW, 15 WHW) and 40 women were on some form of hormonal birth control (oral contraceptives: 10 BLW, 18 WHW; hormonal IUD: 2 BLW, 5 WHW; Nexplanon hormonal implant: 2 BLW, 0 WHW; Depo‐Provera injections: 1 BLW, 1 WHW; vaginal ring: 0 BLW, 1 WHW). On the day of testing, 25 naturally cycling participants reported that they were currently menstruating (11 BLW, 14 WHW) and 21 participants on oral contraceptives reported that they were currently taking their placebo pills (3 BLW, 18 WHW). No differences were observed for educational attainment between groups (*P *> 0.22).

**TABLE 1:** Participant characteristics.

Characteristic	BLW	WHW	*P*
*n*	44	40	—
Age (years)	22 ± 3	22 ± 3	0.96
BMI (kg/m^2^)	24 ± 4	24 ± 2	0.59
Body fat (%)	30 ± 8	28 ± 5	0.13
Parent history of HTN	20 (45)	12 (30)	0.18
Fasting clinical blood values			
Total cholesterol (mg/dl)	164 ± 28	164 ± 27	0.97
HDL cholesterol	63 ± 12	61 ± 15	0.50
LDL cholesterol	89 ± 26	86 ± 22	0.63
Triglycerides	57 ± 20	78 ± 29	<0.001*
Glucose (mg/dl)	85 ± 6	86 ± 7	0.37

Data are presented as means ± SD for continuous variables or *n* (%) for categorical variables. An independent samples *t*‐test was used to assess group differences in continuous variables and Fisher's exact test was used to assess group differences in categorical variables (*n* = 84; 44 BLW, 40 WHW). *P < 0.05. BLW, Black women; BMI, body mass index; HDL, high density lipoprotein; HTN, hypertension; LDL, low density lipoprotein; WHW, White women.

**FIGURE 1:**
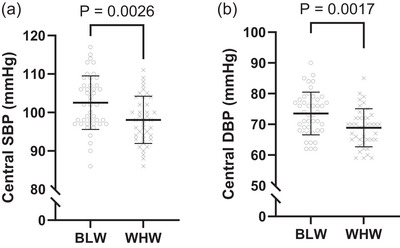
Central systolic (SBP; a) and diastolic (DBP; b) blood pressure displayed between Black women (BLW; ○) and White women (WHW; ×). Independent samples *t*‐tests were used to assess group differences (*n* = 84; 44 BLW, 40 WHW).

**FIGURE 2:**
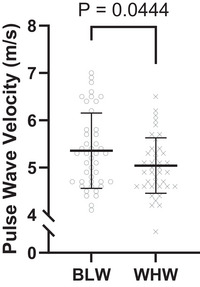
Carotid‐femoral pulse wave velocity displayed between Black women (BLW; ○) and White women (WHW; ×). Independent samples *t*‐tests were used to assess group differences (*n* = 84; 44 BLW, 40 WHW).

All haemodynamic variables were normally distributed except central pulse pressure, augmentation pressure, reflected pulse height and reflection magnitude. Peripheral and central haemodynamic parameters obtained from the SphygmoCor Xcel are displayed in Table 2. Mean systolic and diastolic pBP were higher among BLW. In addition, most central haemodynamic variables including mean systolic and diastolic cBP were higher among BLW (Figure 1), although central pulse pressure, forward pulse height and reflected pulse height were not different between groups. Central artery stiffness assessed as cf‐PWV was higher among BLW (Figure 2).

**TABLE 2:** Pulse wave analysis and pulse wave velocity.

	**BLW**	**WHW**	***P***	**Cohen's *d***
**Peripheral haemodynamics**				
Systolic pBP (mmHg)	115 ± 7	112 ± 7	0.04*	0.43
Diastolic pBP (mmHg)	73 ± 7	68 ± 6	<0.01*	0.77
Peripheral pulse pressure (mmHg)	42 ± 5	44 ± 5	0.19	0.40
Peripheral MAP (mmHg)	87 ± 7	83 ± 6	<0.01*	0.61
**Central haemodynamics**				
Systolic cBP (mmHg)	103 ± 7	98 ± 6	<0.01*	0.77
Diastolic cBP (mmHg)	74 ± 7	69 ± 6	<0.01*	0.77
Central pulse pressure (mmHg)	29 ± 4	29 ± 3	0.76	0.00
Pulse pressure amplification	1.46 ± 0.12	1.46 ± 0.07	0.20	0.00
Central MAP (mmHg)	86 ± 7	80 ± 6	<0.01*	0.92
Heart rate (bpm)	68 ± 11	60 ± 10	<0.01*	0.76
AP (mmHg)	3 ± 4	1 ± 2	0.02*	0.63
AIx	11 ± 11	5 ± 8	0.01*	0.62
AIx75	8 ± 11	0 ± 10	<0.01*	0.76
Forward pulse height (mmHg)	24 ± 3	24 ± 2	0.83	0.00
Reflected pulse height (mmHg)	12 ± 2	11 ± 2	0.06	0.50
Reflection magnitude (%)	48 ± 6	45 ± 6	0.04*	0.50
**Arterial Stiffness**				
cf‐PWV (m/s)	5.4 ± 0.8	5.0 ± 0.6	0.04*	0.57
PWV distance (mm)	412 ± 53	404 ± 47	0.44	0.16
Pulse transit time (ms)	78 ± 9	80 ± 8	0.26	0.23

Data are presented as means ± SD. An independent samples *t*‐tests (parametric) or Mann‐Whitney *U*‐test (non‐parametric) was used to assess group differences (*n* = 84; 44 BLW, 40 WHW). *P < 0.05. AIx, augmentation index; AIx75, augmentation index normalized to a HR of 75 beats per minute; AP, augmentation pressure; BLW, Black women; bpm, beats per minute; cBP, central blood pressure; cf‐PWV, carotid‐femoral pulse wave velocity; MAP, mean arterial pressure; pBP, peripheral blood pressure; PWV, pulse wave velocity; WHW, White women.

## 4 DISCUSSION

We report racial differences in pBP, central haemodynamics, and central arterial stiffness between young, apparently healthy, non‐hypertensive BLW and WHW. Specifically, BLW demonstrated higher pBP, cBP, AIx, AP, RM and cf‐PWV. These findings highlight the importance of considering central haemodynamics and arterial stiffness, in addition to pBP, as promising complementary targets for the management of CVD risk in young BLW.

### 4.1 Racial differences in peripheral BP

Higher systolic and diastolic pBP were observed among young BLW compared with WHW. This finding is consistent with substantial prior evidence. Specifically, BLW have the highest total prevalence of arterial HTN at 58%, which is over 15% higher than women of other races in the United States (Martin et al., 2024). Racial differences in pBP and HTN rates emerge in young adulthood (Shen et al., 2017; Vatsa et al., 2021). Further, the cumulative incidence of HTN from young adulthood to middle age is significantly higher among BLW, independent of pBP values in young adulthood (Thomas et al., 2018). Current clinical recommendations indicate normal systolic pBP as <120 mmHg (Whelton et al., 2018); however, recent findings suggest that women experience increased CVD risk at systolic pBP >100 mmHg compared to systolic pBP <100 mmHg, while the risk is not increased for men until systolic pBP >130 mmHg (Ji et al., 2021). Thus, young non‐hypertensive BLW may experience exacerbated CVD risk at pBP values well below the currently recommended clinical targets.

### 4.2 Racial differences in central haemodynamics

Systolic and diastolic cBPs were elevated among BLW compared with WHW by an average of 5 mmHg in our study. Given that every 10 mmHg increase in systolic cBP is significantly associated with 18% higher risk of cardiovascular‐related mortality among adults (Li, Gao et al., 2024), these results are clinically meaningful. We also observed racial differences in arterial waveforms and wave reflections such that AP, AIx, AIx75 and RM were higher among BLW. Separation of arterial waveforms and wave reflections is an important aspect of central haemodynamics. For example, AP (i.e., augmentation of central pressure) is the amount of pressure added to the systolic pressure peak generated by the reflected wave arriving during systole, thus adding to the amplitude of the forward pressure wave (Nelson et al., 2010). The ratio of AP to central pulse pressure, that is, AIx, is a measure of the enhancement of central aortic pressure by the reflected pulse wave (Nelson et al., 2010). Of note, AIx is highly influenced by heart rate (Wilkinson et al., 2000), and we observed higher heart rate among BLW; still, AIx normalized to a heart rate of 75 bpm (AIx75) remained higher among BLW. Further, in exploratory *post hoc* analyses from the current study, statistical covarying for participant resting HR (Stoner et al., 2014) did not alter the association between race and AIx (data not shown). Despite no racial differences in forward pulse height and reflected pulse height, RM, AIx and AP remained higher among BLW. This suggests a change in the interactions and meeting times of the forward and reflected waveforms and that the contribution of the reflected waveform to afterload may be greater among BLW (Li, Gu et al., 2017).

Increased cBP among young BLW due to altered wave reflection may exacerbate CVD risk in this population as increased cBP has been shown to more strongly relate to target organ damage, vascular and cardiac hypertrophy, extent of atherosclerosis, and cardiovascular events than pBP (Roman et al., 2007; Wang et al., 2009). Importantly, our findings in young women extend findings from previous work by Heffernan et al. (2008) which reported increased cBP and AIx among young Black men compared with White men. Of note, Heffernan et al. (2008) also reported attenuated microvascular function among Black men which they attributed to upstream vascular stiffness. Our lab has previously reported attenuated microvascular function among young BLW compared with WHW (D'Agata et al., 2021, 2021), further suggesting a link between upstream vascular structure and downstream vascular function.

### 4.3 Racial differences in arterial stiffness

BLW had higher cf‐PWV compared with WHW. cf‐PWV is considered the gold‐standard for the measurement of large elastic artery stiffness (Hwang et al., 2014), which has been shown to contribute to elevations in cBP by shifting the return of reflected waves to an earlier time during systole (Laurent, 2012). Accordingly, an increase in arterial stiffness has been shown to have a larger impact on reflected wave amplitude than forward wave amplitude and augments aortic pressure in systole (Nichols et al., 2008), as observed in the present study. Collectively, our findings affirm that increased arterial stiffness is likely contributing to the greater AP, AIx, reflection magnitude, and as a result, cBP in BLW. Previous work demonstrates increased cf‐PWV in BLW compared with WHW as early as young adulthood, independent of pBP (Schutte et al., 2020). Interestingly, additional exploratory analyses from the present study indicate a moderate–strong positive association between pBP and PWV (data not shown), suggesting that arterial stiffness may also be influencing pBP or vice versa. Arterial stiffness is positively associated with atherosclerosis throughout the vascular tree (Palombo & Kozakova, 2016), and clinically a 1 m/s increase in aortic PWV is associated with an ∼15% increase in CVD events and mortality (Vlachopoulos et al., 2010). Further, a study conducted in normotensive 30‐ to 45‐year‐old adults found that PWV was an independent predictor of incident hypertension 4 years later and that a relatively small 1‐SD increase in PWV was associated with a 2.75–2.95 mmHg increase in systolic BP (Koivistoinen et al., 2018). Thus, early detection of arterial stiffening may be especially relevant for young BLW to mitigate elevations in pBP and cBP, and future cardiovascular burden.

### 4.4 Clinical relevance

Although pBP is often the main clinical target (Sharman, 2023), non‐invasive devices for assessing cBP are increasingly accessible and are now used in the management of HTN (Cheng et al., 2020). Evidence suggests a discrepancy between pBP and cBP responses during pharmacological treatment depending on the class of antihypertensive medication used (e.g., beta‐blockers vs. dihydropyridine), further highlighting the need for assessment of both pBP and cBP (Manisty & Hughes, 2013). However, whether these observations hold true in BLW remains to be determined and underscores the need for investigation into the sensitivity and prognostic utility of central haemodynamics in predicting future CVD risk in BLW. Additionally we also observed higher arterial stiffness among BLW, but whether this is a cause or a consequence of increased arterial pressures could not be determined due to this study's cross‐sectional observational design. Importantly, answering such questions would be a critical step towards ensuring the efficacy of blood pressure targeted therapeutics in this at‐risk group, given their potential to differentially effect pBP versus cBP, and thus, cardiovascular outcomes.

### 4.5 Conclusions and future directions

In this study we observed higher pBP, altered central haemodynamics, and increased arterial stiffness among healthy, non‐hypertensive, young adult BLW compared with WHW. Importantly, no differences were observed in participant characteristics, and thus groups were appropriately compared. Future work should explore the prognostic utility of central haemodynamics and arterial stiffness in BLW, which may emerge as intervention targets for the prevention and management of CVD. Identifying underlying mechanisms including social, behavioral and lifestyle factors that may differ among races is also warranted. Lastly, future research should evaluate racial differences in the trajectory of pBP, central haemodynamics, arterial stiffness and vascular function in tandem starting in young adulthood to better understand the magnitude of these measures on racial differences in future cardiovascular outcomes.

## AUTHOR CONTRIBUTIONS

Michele N. D'Agata, Elissa K. Hoopes, Freda Patterson, and Melissa A. Witman contributed to the conception or design of the work. All authors contributed to the acquisition, analysis or interpretation of data for the work, and drafting the work or revising it critically for important intellectual content. All authors approved the final version of the manuscript and agree to be accountable for all aspects of the work in ensuring that questions related to the accuracy or integrity of any part of the work are appropriately investigated and resolved. All persons designated as authors qualify for authorship, and all those who qualify for authorship are listed.

## CONFLICT OF INTEREST

No conflicts of interests, financial or otherwise, are reported.

## Data Availability

The data that support the findings of this study are fully available from the corresponding author to The Journal upon reasonable request.
